# Minute Ventilation/Carbon Dioxide Production Slope Could Predict Short- and Long-Term Prognosis of Patients After Acute Decompensated Heart Failure

**DOI:** 10.3390/life14111429

**Published:** 2024-11-06

**Authors:** Sheng-Hui Tuan, I-Ching Huang, Wei-Chun Huang, Guan-Bo Chen, Shu-Fen Sun, Ko-Long Lin

**Affiliations:** 1 Institute of Allied Health Sciences, College of Medicine, National Cheng Kung University, Tainan 701, Taiwan; ta8101029@gs.ncku.edu.tw; 2 Department of Rehabilitation Medicine, Cishan Hospital, Ministry of Health and Welfare, Kaohsiung 842, Taiwan; 3 Department of Physical Medicine and Rehabilitation, School of Medicine, College of Medicine, Kaohsiung Medical University, Kaohsiung 807, Taiwan; 4 Department of Physical Medicine and Rehabilitation, Kaohsiung Medical University Hospital, Kaohsiung 807, Taiwan; 5 Department of Physical Medicine and Rehabilitation, Kaohsiung Municipal Ta-Tung Hospital, Kaohsiung 801, Taiwan; ichinghuang1202@gmail.com; 6 Department of Critical Care Medicine and Cardiology Center, Kaohsiung Veterans General Hospital, Kaohsiung 813, Taiwan; paper10674@gmail.com; 7 School of Medicine, College of Medicine, National Yang Ming Chiao Tung University, Taipei Campus, Taipei 112, Taiwan; pj73010@gmail.com; 8 Department of Internal Medicine, Kaohsiung Armed Forces General Hospital, National Defense Medical Center, Kaohsiung 802, Taiwan; tanwein@gmail.com; 9 Department of Physical Medicine and Rehabilitation, Kaohsiung Veterans General Hospital, Kaohsiung 813, Taiwan; 10 Department of Physical Medicine and Rehabilitation, Kaohsiung Municipal Siaogang Hospital, Kaohsiung 812, Taiwan

**Keywords:** cardiopulmonary exercise testing, heart failure, oxygen uptake efficiency slope, phase I cardiac rehabilitation, VE/VCO2 slope

## Abstract

(1) Background: Heart failure (HF) leads to functional disability and major cardiovascular events (MACEs). Cardiopulmonary exercise testing (CPET) is the gold standard for assessing aerobic capacity and prognostic stratification. This study aimed to evaluate the predischarge CPET variables in patients with acute decompensated HF and identify the submaximal CPET variables with prognostic value. (2) Methods: A retrospective cohort study was conducted at a tertiary center in Taiwan. Patients surviving their first episode of decompensated HF and undergoing predischarge CPET (February 2017 to January 2023) were analyzed. Follow-up was conducted until a MACE or administrative censoring (up to 5 years). Cox regression identified the significant predictors of MACE. (3) Results: The study included 553, 485, and 267 patients at the 3-month, 1-year, and 5-year follow-ups, respectively. MACE rates were 15.0%, 34.2%, and 50.9%. The VE/VCO2 slope was a significant predictor of MACE at all intervals. A VE/VCO2 slope >38.95 increased the risk of MACE by 2.49-fold at 3 months and 1.81-fold at 1 year (both *p* < 0.001). A slope > 37.35 increased the 5-year MACE risk by 1.75-fold (*p* = 0.002). (4) Conclusions: The VE/VCO2 slope is a significant submaximal CPET predictor of MACE in patients post-acute decompensated HF for both short- and long-term outcomes.

## 1. Introduction

Heart failure (HF), a multifaceted condition characterized by diverse clinical manifestations and consequential functional impairments, has progressed as a global health crisis, affecting an estimated 26 million individuals globally [1]. HF is categorized based on the left ventricular ejection fraction (LVEF) into the following three sub-types: HF with reduced ejection fraction (HFrEF) (LVEF of ≤40%), HF with preserved ejection fraction (HFpEF) (LVEF of ≥50%), and HF with midrange ejection fraction (HFmrEF) (LVEF of 41–49%) [2]. The survival rate of HF has improved due to the advancements in medical interventions and diagnostic technologies [3]. The prognostic outcomes, both in the acute phase and over long-term follow-up, demonstrate variability across these subtypes and among different ethnic groups [2].

Patients with HF frequently experience exercise intolerance, marked by a reduced capacity for physical activity. The causes of this intolerance in HF are multifaceted, including impaired cardiac and pulmonary reserves and diminished skeletal muscle perfusion and functionality [4]. Evaluating a patient’s exercise tolerance is crucial because exercise intolerance directly diminishes the quality of life and increases mortality risk, particularly against the backdrop of extended survival in patients with HF [4]. A range of methods is used to evaluate the degree of exercise intolerance affecting the functional capacity in patients with HF. These methods include the New York Heart Association functional classification, quality of life assessments, electrocardiogram (ECG) stress testing, the six-minute walk test, and cardiopulmonary exercise testing (CPET). CPET, which specifically measures peak oxygen uptake (peak VO2), has been regarded as the gold standard for predicting event-free survival in patients with HF across all classifications for over 25 years [5,6].

Obstacles, such as peripheral muscle fatigue, dyspnea, and significant cardiac alterations, frequently prevent patients from reaching maximal exercise efforts despite peak VO2 status as the criterion standard in HF evaluations [7]. This is particularly true for patients with HFpEF, who frequently fail to achieve peak VO2 [8], not to mention those with HFpEF [9] and HFmrEF [10]. Additionally, patients with acute decompensated HF (defined as newly onset or worsening symptoms and signs of HF) following treatment usually remain in markedly reduced exercise capacity, causing early termination when performing CPET [11]. Hence, submaximal CPET parameters have become increasingly relevant for evaluating patients with cardiovascular disorders. Indices, such as the anaerobic threshold (AT), the slope of the association between minute ventilation and carbon dioxide production (VE/VCO2 slope), work efficiency, and the oxygen uptake efficiency slope (OUES), are strongly correlated with cardiac function [12,13]. Among these, the impaired VE/VCO2 slope has been proven as a complementary, and frequently superior, event-free survival predictor compared to peak VO2 in patients with HFrEF [14] and HFpEF [15]. The VE/VCO2 slope is increased in most patients with HF [15], with this increase inversely associated with cardiac output at peak exercise, largely because of reduced pulmonary perfusion [16]. However, a majority of the previously mentioned studies have tracked patients with HF for 1–2 years, leaving the long-term prognostic effect of the VE/VCO2 slope among patients with HF largely unexplored.

The 2021 guidelines from the European Society of Cardiology on diagnosing and treating both acute and chronic HF emphasize the positive effect of exercise training on patients with HF. Notable advantages of such training include an improved ability to exercise, better health-related quality of life, and decreased hospital readmission frequency [2]. Kaneko et al. have determined several prognostic benefits related to the early initiation of phase I cardiac rehabilitation in patients with acute decompensated HF [17]. CPET provides physiatrists with crucial information about exercise tolerance and potential risks, enabling them to prescribe optimal exercise intensities for patients with acute decompensated HF. The significance of cardiac rehabilitation in patients with chronic HF is widely recognized, but its application in the acute HF setting remains underexplored due to safety concerns [4] and only a limited number of hospitals, including tertiary centers, perform predischarge CPET for patients with acute decompensated HF [18]. Moreover, the long-term prognostic value of submaximal CPET variables in the predischarge status of patients with acute decompensated HF remains uncertain. Therefore, our current study aimed to explore the short- and long-term prognostic values of predischarge CPET variables, specifically the VE/VCO2 slope, to provide valuable information for physicians managing patients post-acute decompensated HF.

## 2. Materials and Methods

### 2.1. Study Design and Participants

This cohort study included patients who were hospitalized for acute decompensated HF at a tertiary medical center in Southern Taiwan from February 2017 to January 2023. Patients had to meet the following criteria to be eligible for the study: (1) be 20 years of age or older; (2) have been diagnosed as heart failure previously and admitted for first acute decompensated HF; (3) have completed a CPET before discharge; and (4) possess a comprehensive record of transthoracic echocardiographic examination and a standard 12-lead electrocardiogram (ECG). Exclusion criteria were patients considered too frail for CPET, encompassing those with cognitive impairment, neuromuscular disorders, dependence on a ventilator, severe pulmonary disorders necessitating oxygen therapy, prolonged bedridden status of >3 months, or those with late-stage cancer whose life expectancy was expected to be less than one year. Additionally, this study excluded patients with incomplete data, lacking CPET records, or without consistent medical follow-ups for at least three months after their acute decompensated HF event.

We conducted a retrospective analysis of the demographic, clinical, and angiographic information from the patients’ medical records, including details on medical history, medication use, smoking status, and body mass index. The follow-up period ranged from the initial hospital admission for acute decompensated HF to the first major adverse cardiac event (MACE) or until administrative censoring, which extends up to five years. The cardiology department at the medical center provided outpatient follow-up care. MACEs were determined as hospitalizations for HF requiring emergency intravenous diuretics, coronary revascularization, myocardial infarction, cerebral vascular accidents, or cardiovascular death [19], and were documented at 3 months, 1 year, and 5 years post-discharge. Cardiologists confirmed MACE occurrence through medical records. Patients experiencing MACEs were categorized into the adverse event group, whereas those without MACEs during each follow-up period (within 3 months, 1 year, and 5 years) were classified in the adverse event-free group. The Institutional Review Board of Kaohsiung Veterans General Hospital approved the study (VGHKS17-CT11-11), and all the participants, who were briefed about the purpose of the CPET and the study’s objectives, provided informed consent.

### 2.2. Cardiopulmonary Exercise Testing

Each patient underwent a symptom-limited, progressive exercise test with a leg ergometer, flow module, gas analyzer, and ECG monitor [Metamax 3B, Cortex Biophysik GmbH Co., Leipzig, Germany]. The protocol increased the workload incrementally by 10 W/min [20]. Testing was halted upon the emergence of subjectively unbearable symptoms, including severe shortness of breath, chest pain, severe dizziness, excessive fatigue, physical instability, or excessive pallor, following the criteria set by the American College of Sports Medicine [20], or when patients could no longer continue, or if they reached a submaximal endpoint. This endpoint was defined as achieving work at ≥75 W/min, the peak oxygen consumption of ≥5 metabolic equivalents (METs), the peak heart rate of ≥70% of the age-predicted maximum, or a respiratory exchange ratio of ≥1.1. These criteria, adapted and modified by Lin et al. [21], have been effectively used in our hospital for patients with acute myocardial infarction and HF, proving suitable for this population. All patients underwent the CPET under the supervision of a physiatrist with >10 years of experience (K.-L.L.).

We continuously measured oxygen consumption (VO2) and carbon dioxide production (VCO2) on a breath-by-breath basis during the CPET. Additionally, the AT, respiratory rate, and other data derived, such as the respiratory exchange ratio and the VE/VCO2 slope, were evaluated. The AT is determined by a noticeable increase in the VCO2–VO2 slope [22]. Peak VO2 represents the highest value of oxygen uptake recorded during the test, whereas peak METs are calculated as the peak VO2 divided by a standard value of 3.5 mL/kg/min. The percentage of peak VO2 relative to its predicted value (predicted peak VO2%) was identified by comparing the measured peak METs against the predicted values based on Taiwan’s normal standards [23]. The peak rate pressure product was defined as peak systolic blood pressure multiplied by peak heart rate. The VE/VCO2 slope was calculated from the start of the test up to just beyond the AT [24]. The OUES was calculated with the graphic slope (a) of the following equation: VO2 = a log (VE) + b. The OUES was calculated with the total exercise time (OUES 100) [25]. The OUES was normalized by the body surface area (BSA) due to the anthropometric variation [26]. Haycock’s formula was utilized to calculate the BSA [27].

### 2.3. Statistical Analysis

Before conducting any analysis, we confirmed the normality and homogeneity of variance of the data. Categorical variables were analyzed with the Chi-squared test or Fisher’s Exact test as appropriate to differentiate between the outcomes of the adverse event and adverse event-free groups, whereas the independent *t*-test was applied to normally distributed continuous variables, and the Mann–Whitney U test was used for those not following a normal distribution. Receiver operating characteristic curves were plotted to identify the optimal cutoff values for each CPET variable in predicting MACEs at 3 months, 1 year, and 5 years, by identifying the point where the sum of sensitivity and specificity was the highest.

Kaplan–Meier survival curves and the log-rank test were used to compare the incidence of MACEs between the adverse event and the adverse event-free groups. Multivariate Cox regression analysis was conducted to calculate the hazard ratios (HRs) for potential prognostic factors. A two-tailed *p*-value of <0.05 indicated statistical significance. The Statistical Package for the Social Sciences for Windows, version 21.0 (IBM Corp., Armonk, NY, USA) was used for all statistical analyses. The minimum sample size required for our study was identified using an online calculator from the University of California, San Francisco, designed for survival analysis according to the HR regression model (URL: https://sample-size.net/sample-size-survival-analysis/; accessed on 6 March 2024). This calculation accounted for a type I error rate of 0.05 and a power of 0.8, assuming a 1:1 ratio between the exposed group (with MACEs) and the non-exposed group (without MACEs) at the 5-year follow-up, in line with the previous literature [15]. The relative hazard was expected to be 2.0, based on previous studies [15]. Accordingly, the minimum sample size calculated for each group was 74.

## 3. Results

### 3.1. Study Population

A total of 606 patients were referred to our rehabilitation department for predischarge CPET following their initial episode of acute decompensated HF. Of these, 53 patients were excluded from the study due to various reasons: 38 lacked consistent medical follow-ups for a minimum of three months, 6 had missing ECG data, and 7 had incomplete echocardiography records, while 2 had terminal-stage cancer with life expectancy less than 1 year (1 with lung cancer, 1 with colorectal cancer). Consequently, the study proceeded with 553 patients for the 3-month analysis. For the 1-year follow-up, 485 patients remained eligible for inclusion, as 68 (32 of 68 dead) patients experienced their first episode of acute decompensated HF less than 1 year prior to the study. At the 5-year mark, 267 patients were analyzed, with 218 (26 of 218 dead) patients having their initial acute decompensated HF episode less than 5 years before the commencement of this study. Regarding the causes of acute decompensated HF, 179 cases (32.4%) were attributed to acute coronary syndrome, while the remaining cases were due to infection, arrhythmia, valvular dysfunction, and hypertensive pulmonary edema, among other factors.

The mean duration from HF diagnosis to their first admission was 3.2 ± 2.1 years. The time from the initial admission for acute decompensated HF to the predischarge CPET averaged 5.58 ± 2.36 days. We compared the demographic and clinical data between survivors of acute decompensated HF with and without MACEs at 3 months, 1 year, and 5 years (Table 1). There were no significant differences in age, BMI, smoking status, gender, comorbidities, or basic biochemistry profiles, which included measurements of the serum creatinine, total cholesterol, low-density lipoprotein cholesterol, glycated hemoglobin, and N-terminal pro B-type natriuretic peptide (NT-ProBNP), medications, and NYHA classification across the groups. However, there was a notable difference in medication use (Table 1, lower rows) as follows: patients who experienced MACEs had a higher usage of angiotensin receptor–neprilysin inhibitors (ARNI) than those without MACEs at the 3-month mark (*p* = 0.034), and a greater intake of sodium–glucose co-transporter 2 (SGLT2) inhibitors was observed in the MACE group compared to the non-MACE group at 5 years (*p* = 0.021). Moreover, the non-MACE group had a higher percentage of diuretic use (excluding SGLT2 inhibitors) at 3 months, 1 year, and 5 years, compared to the MACE group (all *p* < 0.05). Throughout follow-up, the group with MACEs exhibited a higher death rate (*p*-values: 0.009, <0.001, and 0.005, respectively). Additionally, the group without MACEs showed a higher LVEF than those with MACEs at 1 year (*p* = 0.002), but no significant difference in LVEF was observed between the two groups at the 3-month and 5-year follow-ups (Table 1, mid-row). Notably, although not statistically significant, HF patients who experienced MACEs in our study showed a relatively higher prevalence of COPD at 3 months, 1 year, and 5 years (Table 1, upper third row).

### 3.2. Parameters of Cardiopulmonary Exercise Testing

Among the study’s eligible participants, the mean and median values for peak RER were 1.1 ± 0.1 and 1.1, respectively. A total of 46 participants terminated the CPET prematurely due to various reasons, including dyspnea (*n* = 21), fatigue (*n* = 15), dizziness (*n* = 5), pallor (*n* = 3), and chest tightness (*n* = 2). Comparative analysis of the CPET parameters between the MACE group and the MACE-free group at different follow-up intervals revealed the following:

At the 3-month follow-up, the MACE-free group demonstrated a lower VE/VCO2 slope (*p* = 0.026), higher peak metabolic equivalents (MET) (*p* = 0.010), predicted peak oxygen consumption percentage (predicted peak VO2%) (*p* = 0.038), OUES 100 (*p* = 0.033), OUES 100/BSA (*p* = 0.048), and 6-min walk test (6MWT) distance (*p* = 0.004) compared to the MACE group (Table 2, left column).

At the 1-year follow-up, the MACE-free group exhibited a lower VE/VCO2 slope (*p* = 0.028), higher resting systolic blood pressure (SBP) (*p* < 0.001), resting diastolic blood pressure (DBP) (*p* = 0.008), peak SBP (*p* = 0.002), peak DBP (*p* = 0.024), peak MET (*p* < 0.001), predicted peak VO2% (*p* < 0.001), peak rate–pressure product (PRPP) (*p* = 0.003), OUES 100/BSA (*p* = 0.011), and 6MWT distance (*p* < 0.001) compared to those in the MACE group (Table 2, middle column).

At the 5-year follow-up, the MACE-free group had a lower VE/VCO2 slope (*p* = 0.004), higher resting SBP (*p* = 0.015), peak SBP (*p* = 0.003), peak MET (*p* < 0.001), maximal watt (*p* = 0.015), predicted peak VO2% (*p* < 0.001), PRPP (*p* = 0.008), OUES 100 (*p* = 0.021), OUES 100/BSA (*p* = 0.004), and 6MWT distance (*p* = 0.008) compared to the MACE group (Table 2, right column).

### 3.3. Multivariate Analyses for Identification of Predictors and the Survival Probability

Receiver operating characteristic (ROC) curves were utilized to assess the predictive value of the VE/VCO2 slope, peak MET, predicted peak VO2%, OUES 100/BSA, and the 6-min walk test (6MWT) for the three-month MACEs. The area under the curve (AUC) results in descending order were as follows: VE/VCO2 slope 0.607 (*p* = 0.003); peak MET 0.428 (*p* = 0.043); OUES 100/BSA 0.429 (*p* = 0.047); predicted peak VO2% 0.424 (*p* = 0.033); and 6MWT 0.408 (*p* = 0.010) (Table 3, upper rows).

For predicting one-year MACEs, ROC analysis included the VE/VCO2 slope, resting systolic blood pressure (SBP), resting diastolic blood pressure (DBP), peak SBP, peak DBP, peak MET, predicted peak VO2%, peak rate–pressure product (PRPP), OUES 100/BSA, and 6MWT. The AUC values in descending order were as follows: VE/VCO2 slope 0.583 (*p* = 0.004), peak DBP 0.439 (*p* = 0.033), resting DBP 0.431 (*p* = 0.015), OUES 100/BSA 0.416 (*p* = 0.003), PRPP 0.409 (*p* = 0.001), 6MWT 0.408 (*p* = 0.001), peak SBP, peak MET, and predicted peak VO2% each with AUC values 0.399 (*p* < 0.001) and resting SBP 0.385 (*p* < 0.001) (Table 3, middle rows).

The five-year MACE prediction involved ROC curves for VE/VCO2 slope, resting SBP, peak SBP, peak DBP, peak MET, maximal watt, predicted peak VO2%, OUES 100/BSA, and 6MWT. The corresponding AUC values in descending order were VE/VCO2 slope 0.621 (*p* = 0.001), maximal watt 0.424 (*p* = 0.039), 6MWT distance 0.414 (*p* = 0.021), OUES 100/BSA 0.410 (*p* = 0.016), peak METs AUC 0.416 (*p* = 0.024), predicted peak VO2% 0.399 (*p* = 0.006), peak DBP 0.391 (*p* = 0.003), resting SBP 0.389 (*p* = 0.007), and peak SBP 0.388 (*p* = 0.003) (Table 3, lower rows).

**Figure 1:**
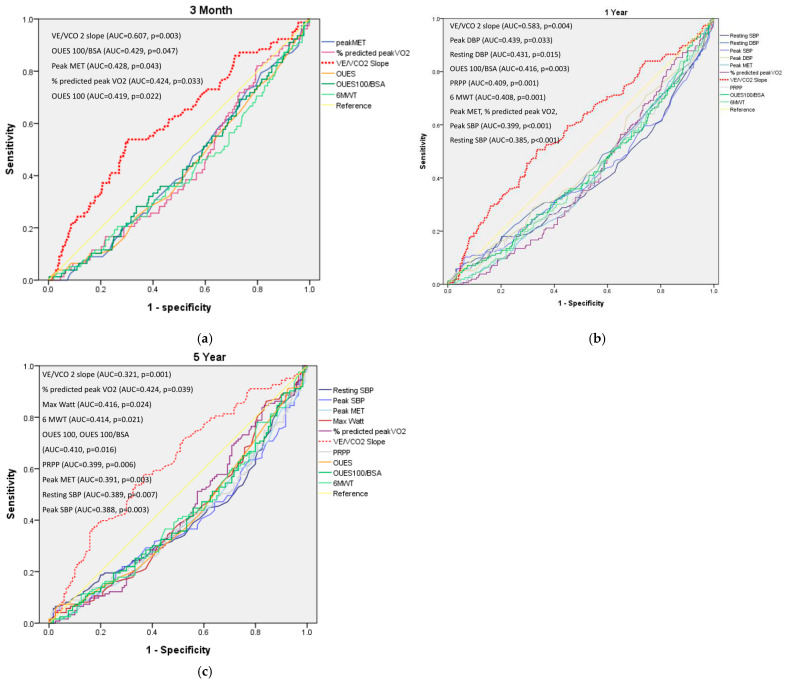
ROC curve of cardiopulmonary exercise testing variables in determination of MACEs of patients survived acute decompensated heart failure at three months (**a**), one year (**b**), and five years (**c**). 6MWT, 6-min walking test; AUC, area under curve; BSA, body surface area; DBP, diastolic blood pressure; MET, metabolic equivalent; OUES 100, oxygen uptake efficiency slope calculated from data of the whole exercise duration; PRPP, peak rate pressure product; SBP, systolic blood pressure; % predicted peak VO2, percentage of measured peak oxygen consumption to estimated peak oxygen consumption.

Each of the above variables of AUC was determined by the maximum sum of sensitivity and specificity (Table 3 and Figure 1).

**Figure 2:**
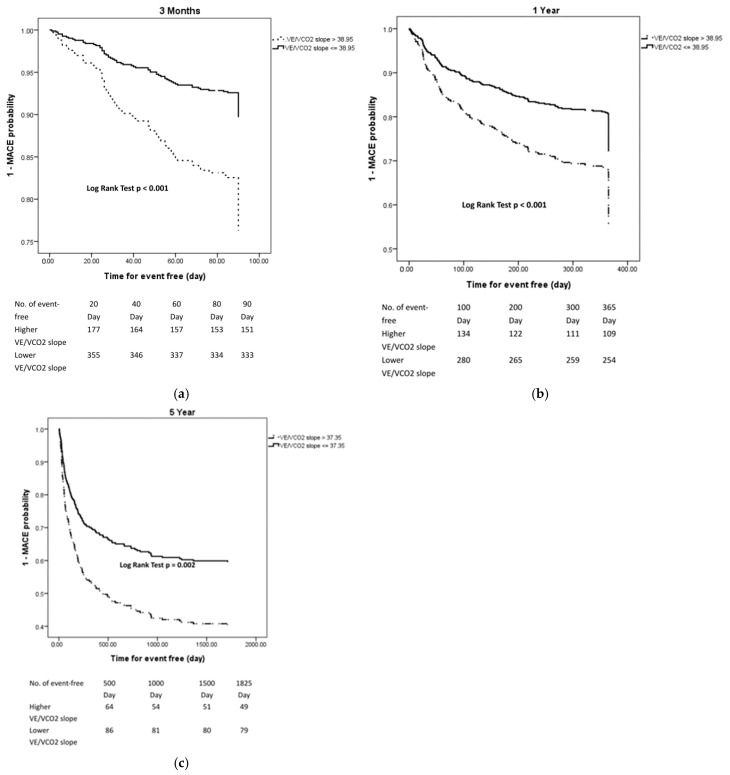
Kaplan–Meier analysis of three-month (**a**), one-year (**b**), and five-year (**c**) major adverse cardiovascular events (MACEs) in patients who survived acute decompensated heart failure with high and low minute ventilation/carbon dioxide production slope (VE/VCO2 slope).

The occurrence of MACEs at three months, one year, and five years post-discharge was 15.0%, 34.2%, and 50.9%, respectively. An AUC value of 0.5 on the ROC analyses signifies that the test’s prediction accuracy is no better than chance, indicating the test lacks discriminative power. Consequently, ROC curves that rise above this threshold demonstrate a test’s capability to distinguish between the patients with and without a specific disease or condition effectively [28]. Therefore, we conducted survival analyses exclusively on variables exhibiting an AUC greater than 0.5, specifically the VE/VCO2 slope during the follow-up periods of three months, one year, and five years, to evaluate their predictive accuracy for MACEs. After adjusting for age and gender, Kaplan–Meier survival analysis and the log-rank test revealed statistically significant associations, indicating that a higher VE/VCO2 slope corresponded with an increased risk of MACEs. Specifically, the HR was 2.488 (*p* < 0.001) at three months, the HR was 1.807 (*p* < 0.001) at one year, and the HR was 1.749 (*p* = 0.002) at five years. The cut-off values identified for the VE/VCO2 slope were 38.95 at both three months and one year and 37.35 at five years, demonstrating its predictive value for MACE risk across these time frames. (Table 4 and Figure 2).

Patients who survived acute decompensated heart failure with higher VE/VCO2 slope showed significantly higher rate of three-month (*p* < 0.001), one-year (*p* < 0.001), and five-year (*p* = 0.002) MACEs than those with a lower VE/VCO2 slope.

## 4. Discussion

Our research reveals that the VE/VCO2 slope serves as a crucial prognostic marker for both the short- and long-term outcomes in patients who have survived acute decompensated HF. Importantly, this study is the first to highlight the significance of the VE/VCO2 slope within the context of phase I cardiac rehabilitation, establishing its value as a predictor of prognosis for up to five years.

The VE/VCO2 slope quantifies the increase in ventilation relative to CO2 production, thereby indicating an enhanced ventilatory drive [29]. A heightened VE/VCO2 slope signifies ventilatory inefficiency, characterized by a disproportionately rapid increase in ventilation (VE) compared to CO2 output (VCO2), accompanied by a significant drop in arterial CO2 levels leading to hypocapnia [30]. Our current findings corroborate the previous research, showing that patients with chronic HF exhibit an elevated exercise ventilatory response, as evidenced by an increased VE/VCO2 slope. This elevation during exercise in HF patients might be attributed to a mix of hyperventilation and augmented dead space ventilation [31]. Additionally, the intensification of ventilatory drive may also result from a heightened activation of the mechano-sensitive j-receptors due to abnormal expansion of the pulmonary vasculature seen in pulmonary congestion [32].

Multiple studies have established that a VE/VCO2 slope exceeding 34, and particularly values above 45, marks patients with HF as higher risk, forecasting a poorer prognosis for individuals with HF [33,34]. Additionally, the VE/VCO2 slope has been independently linked to the total number of HF hospitalizations specifically within the HFrEF cohort [35]. Invasive hemodynamic comparisons have illustrated that the VE/VCO2 slope, when measured during submaximal exercise, accurately mirrors key cardiac and pulmonary pressures, including cardiac output, pulmonary artery pressure, and pulmonary capillary wedge pressure [36]. Our research confirms that the VE/VCO2 slope serves as a crucial predictor of MACEs across short-, intermediate-, and long-term follow-ups in Chinese HF patients. We identified optimal VE/VCO2 slope thresholds for MACE prediction as >38.95 for up to 3 months and 1 year, and >37.35 for up to 5 years in the CHF patient population. Shen et al. observed that for Chinese patients with chronic HF, the VE/VCO2 slope had optimal threshold values of ≥39.3 for predicting cardiac-related mortalities and ≥32.9 for cardiac-related hospitalizations [37]. Similarly, Arena et al. identified an optimal VE/VCO2 slope threshold of ≥32.9 for forecasting cardiac-related hospitalization among American HF patients [38]. In our study, MACEs encompassed re-hospitalizations for HF, coronary revascularization, myocardial infarction, cerebral vascular accidents, and cardiovascular death. Given these considerations, it was reasonable for our findings to indicate a predictive threshold for the VE/VCO2 slope ranging between 32.9 and 39.3, aligning with previous research while taking into account the broader spectrum of adverse outcomes included in our study.

Several studies have demonstrated that an elevated VE/VCO2 slope is linked to increased mortality in patients with HFrEF [16,39]. The utility of the VE/VCO2 slope in HFpEF patients remains a topic of debate. Guazzi et al. reported that, in a cohort of 46 patients, the VE/VCO2 slope (but not peak VO2) was associated with all-cause mortality and hospitalization at 1 year [40]. This group later found that the VE/VCO2 slope, but not peak VO2, could predict cardiac-related death in a sample of 151 HFpEF patients over a median follow-up of 13 months [41]. Conversely, Shafiq et al. identified that peak VO2, but not the VE/VCO2 slope, correlated with all-cause mortality or cardiac transplant in a study of 173 HFpEF patients followed for a median of 5.2 years [42]. Nadruz et al. observed that both VE/VCO2 slope and peak VO2 (absolute or percent of predicted) independently prognosticated outcomes in 195 HFpEF patients over 2 years [35]. Few studies have delved into the prognostic significance of the VE/VCO2 slope or peak VO2 in HFmEF patients. Nadruz et al. suggest that clinical characteristics of HFmEF patients typically lie between those of HFpEF and HFrEF, with the relative risk associated with peak VO2 showing intermediate values across these groups. Meanwhile, the VE/VCO2 slope demonstrated a neutral relationship with incident heart failure hospitalization in HFmEF patients [35]. In contrast, Gong et al. discovered that a higher VE/VCO2 slope is linked to an increased risk of 2-year all-cause mortality and heart failure hospitalization across the entire spectrum of heart failure [43]. Our study’s participants predominantly had HFrEF (52%), followed by HFmEF (28%) and HFpEF (20%). Our findings indicate that the VE/VCO2 slope, but not peak VO2, could predict MACEs across short-, intermediate-, and long-term follow-ups.

CPET serves as a critical and objective tool for evaluating the functional capacity of patients with HF [34]. Identifying submaximal CPET variables with prognostic value is crucial, especially since patients recently recovering from acute decompensation may struggle to reach peak exercise levels. Consistent with prior research, our findings indicate that HF patients without MACEs exhibited higher OUES 100/BSA [11] and achieved longer distances in the 6MWT [39]. Nevertheless, the AUC for these submaximal CPET variables was below 0.5 in our study, indicating limited predictive accuracy. Similarly, the AUC for peak VO2 in our study also fell below 0.5 at the 3-month, 1-year, and 5-year follow-ups, underscoring its limited prognostic utility. Therefore, our analysis underscores the superior prognostic value of the VE/VCO2 slope among these variables, highlighting its significance in the assessment of HF patients. These findings align with numerous studies indicating the superior prognostic value of the VE/VCO2 slope over peak VO2 in HF prognosis [33,34,38]. The VE/VCO2 slope offers a more comprehensive evaluation of the intricate interactions between the pulmonary, cardiac, and peripheral aspects of heart failure [44]. Furthermore, this parameter is less prone to variability and less dependent on patient effort level compared to peak VO2 [16], making it a more stable indicator of cardiovascular health. Within the context of the REVIVAL study, which analyzed exercise parameters in advanced HF patients, the VE/VCO2 slope emerged as the most reliable and significant MET predictor of adverse outcomes, including the need for mechanical circulatory support implantation, transplantation, or death within a year [45].

Last but not the least, we would like to emphasize that the use rates of ARNI and SGLT2 inhibitors in our study were relatively low (ARNI 47.9% and SGLT2 inhibitors 21.5%), likely due to the timing of our first follow-up between 2017 and 2018, before the 2021 guidelines recommended these medications. Although recent real-world data show gradual adoption, the Get With The Guidelines—Heart Failure registry (July 2021 to June 2022) reported that only 20.2% of eligible heart failure patients were discharged with SGLT2 inhibitors, reflecting limited uptake despite guideline endorsement [46]. Conversely, data from the Swedish Heart Failure Registry (November 2020 to August 2022) indicated an increase in SGLT2 inhibitor use from 20.5% to 59.0% over time; ARNI prescriptions also rose, with a Japanese study showing a 43.9% rate between July 2021 and September 2022. Thus, it is important for readers to interpret our data in the context of real-world clinical practice and evolving guidelines.

To the best of our knowledge, this study is the first investigation into the prognostic utility of the VE/VCO2 slope measured in pre-discharge CPET among Chinese HF patients over a follow-up period extending up to 5 years. In our cohort, the average duration between initial admission for acute decompensated HF and the pre-discharge CPET was 5.58 ± 2.36 days. In comparison, Yan et al.’s research on 224 HFpEF patients, with an average follow-up of 30 months, demonstrated that the VE/VCO2 slope, not peak VO2, was linked to all-cause mortality once adjusted for clinical variables and BNP levels [46]. Similarly, Shafiq et al. studied 173 HFpEF patients over a median follow-up of 5.2 years (IQR 3.4–7.9 years), finding that peak VO2, but not the VE/VCO2 slope, was associated with all-cause mortality or cardiac transplant [42]. Notably, the CPET in these studies was conducted post-admission for HF, corresponding to phase II cardiac rehabilitation, unlike the phase I rehabilitation setting of our study. Cardiac rehabilitation in chronic HF patients offers multiple benefits to cardiopulmonary fitness, including enhanced exercise capacity, autonomic function, endothelial function, reduced depressive symptoms, and the facilitation of left ventricular reverse remodeling [17]. The early initiation of phase I cardiac rehabilitation in hospitalized patients with acute decompensated HF has been shown to improve short-term prognostic outcomes significantly, such as lowering in-hospital mortality, reducing the length of hospital stays, and decreasing the 30-day readmission rate [17]. Our findings underscore the importance of commencing cardiac rehabilitation and individualized exercise prescriptions based on CPET-determined cardiopulmonary fitness before discharging HF patients, potentially guiding both phase I and phase II cardiac rehabilitation strategies.

Last but not the least, we would like to emphasize that the use rates of ARNI and SGLT2 inhibitors in our study were relatively low (ARNI 47.9% and SGLT2 inhibitors 21.5%), likely due to the timing of our first follow-up between 2017 and 2018, before the 2021 guidelines recommended these medications. Although recent real-world data show gradual adoption, the Get With The Guidelines—Heart Failure registry (July 2021 to June 2022) reported that only 20.2% of eligible heart failure patients were discharged with SGLT2 inhibitors, reflecting a limited uptake despite guideline endorsement [47]. Conversely, data from the Swedish Heart Failure Registry (November 2020 to August 2022) indicated an increase in SGLT2 inhibitor use from 20.5% to 59.0% over time [48], and ARNI prescriptions also rose, with a Japanese study showing a 43.9% rate between July 2021 and September 2022 [49].

This study acknowledges several limitations. First, recruitment was limited to a single medical center in Southern Taiwan, which may have affected the generalizability of the findings to wider populations. The study predominantly enrolled male patients, reflecting the demographic characteristics of the center. Second, although the sample size surpassed the minimum requirement and our research is the first study to track MACEs in patients surviving acute decompensated HF for up to 5 years, the sample size was still relatively small. This limitation could potentially affect the statistical power of the findings. Additionally, due to the limited numbers of patients with HFpEF and HFmrEF, a subgroup analysis to assess the prognostic value differences of the CPET variables among diverse HF populations was not performed. Future research should focus on these specific HF populations. Third, by concentrating on individuals who survived their first episode of acute decompensated HF following successful treatment, the study may have introduced selection bias. This focus limits the broader applicability of the results to those with more severe conditions. Fourth, certain COPD medications, such as LABAs and LAMAs, are associated with increased cardiovascular risk. Although we did not find a significant difference in the prevalence of COPD between the MACE-free and MACE groups at 3 months, 1 year, and 5 years, it would be beneficial to document and compare COPD treatments among our participants [50].

## 5. Conclusions

In conclusion, our study suggests that the VE/VCO2 slope may hold significant value as a prognostic marker for patients recovering from acute decompensated HF. Our findings indicate that the VE/VCO2 slope can potentially predict MACE risk in three months, one year, and even up to five years. Identifying submaximal predictors such as the VE/VCO2 slope may be particularly valuable given the challenges HF survivors face in reaching peak exertion during the CPET. This highlights the role that the CPET could play in risk stratification and individualized exercise prescription within cardiac rehabilitation programs for the acute HF population.

## Figures and Tables

**Table 1:** Participant characteristics according to the outcome group.

Variables		Within 3 Months			Within 1 Year			Within 5 Years		
		Adverse Event-Free Group (*n* = 470)	Adverse Event Group (*n* = 83)	*p* Value ^a^	Adverse Event-Free Group (*n* = 319)	Adverse Event Group (*n* = 166)	*p* Value ^a^	Adverse Event-Free Group (*n* = 131)	Adverse Event Group (*n* = 136)	*p* Value ^a^
Sex—Female, *n* (%)		111 (23.6%)	18 (21.7%)	0.701	80 (25.1%)	33 (19.9%)	0.199	31 (23.7%)	30 (22.1%)	0.755
Age (years)		59.8 ± 15.4	62.5 ± 14.8	0.131	61.4 ± 15.0	63.4 ± 15.8	0.172	66.9 ± 15.3	68.7 ± 15.5	0.341
Duraiton from diagnosis of heart failure to first admission		3.14 ± 2.1	3.54 ± 2.9	0.134	4.11 ± 2.0	4.37 ± 3.3	0.282	7.90 ± 3.2	8.49 ± 4.3	0.206
BMI (kg/m^2^)		25.6 ± 6.1	24.4 ± 4.9	0.094	25.4 ± 6.3	24.9 ± 5.5	0.341	25.5 ± 6.6	24.9 ± 5.4	0.351
NYHA, *n* (%)	I	4 (0.9%)	3 (3.6%)	0.241	5 (1.6%)	2 (1.2%)	0.445	4 (3.1%)	1 (0.7%)	0.462
	II	113 (24.0%)	18 (21.7%)		77 (24.1%)	33 (19.9%)		28 (21.4%)	28 (20.6%)	
	III	319 (67.9%)	58 (69.9%)		214 (67.1%)	125 (75.3%)		87 (66.4%)	101 (74.3%)	
	IV	34 (7.2%)	4 (4.8%)		23 (7.2%)	6 (3.6%)		12 (9.1%)	6 (4.4%)	
Smoker, *n* (%)		59 (63.4%)	14 (70.0%)	0.578	32 (60.4%)	41 (68.3%)	0.377	24 (57.1%)	49 (69.0%)	0.202
Cause of heart failure	DCM	51 (10.9%)	6 (7.2%)	0.132	30 (9.4%)	18 (10.8%)	0.502	7 (5.3%)	15 (11.0%)	0.065
	CAD	147 (31.3%)	32 (38.6%)		101 (31.7%)	53 (31.9%)		33 (25.2%)	47 (34.6%)	
	Valvular disease	79 (16.8%)	7 (8.4%)		59 (18.5%)	22 (13.3%)		25 (19.1%)	18 (13.2%)	
	others	193 (41.1%)	38 (45.8%)		129 (40.4%)	73 (44.0%)		66 (50.4%)	56 (41.2%)	
Comorbidities	Hypertension	321 (68.3%)	56 (67.5%)	0.917	233 (73.0%)	105 (63.30%)	0.041 *	94 (71.8%)	94 (69.1%)	0.698
	Diabetes	196 (41.7%)	37 (44.6%)	0.702	139 (43.6%)	61 (36.7%)	0.175	58 (44.3%)	51 (37.5%)	0.313
	Dyslipidemia	208 (44.3%)	40 (48.2%)	0.517	140 (43.9%)	72 (43.4%)	0.872	55 (42.0%)	63 (46.3%)	0.541
	COPD	30 (6.4%)	2 (2.4%)	0.274	23 (7.2%)	7 (4.2%)	0.242	11 (8.4%)	4 (2.9%)	0.117 ^b^
Biochemistry data	Cr (umol/L)	75.4 ± 16.1	77.1 ± 11.7	0.655	73.2 ± 14.3	77.9 ± 13.6	0.079	72.2 ± 12.9	77.8 ± 18.6	0.088
	TC (mmol/L)	5.0 ± 0.6	5.2 ± 0.5	0.130	4.9 ± 0.8	5.1 ± 0.6	0.161	5.0 ± 0.6	5.1 ± 0.7	0.407
	LDL (mmol/L)	2.7 ± 0.6	2.9 ± 0.5	0.198	2.7 ± 0.7	2.8 ± 0.6	0.258	2.7 ± 0.7	2.8 ± 0.7	0.444
	HbA1C (%)	6.2 ± 1.4	6.4 ± 1.6	0.532	6.1 ± 1.3	6.3 ± 1.4	0.399	6.1 ± 1.4	6.3 ± 1.6	0.415
	NT-proBNP (pg/mL)	6624 ± 28,377	10,194 ± 27,080	0.440	8028 ± 33,965	8392 ± 21,884	0.933	17,948 ± 57,080	10,828 ± 26,330	0.435
Echocardiography	LVEF (%)	39.7 ± 11.2	37.9 ± 10.3	0.215	39.6 ± 11.2	36.1 ± 10.2	0.002 *	34.7 ± 9.0	33.5 ± 10.4	0.397
	HFrEF	237 (50.4%)	52 (62.7%)	0.102	166 (52.0%)	108 (65.1%)	0.033 *	91 (69.5%)	102 (75.0%)	0.431
	HFmrEF	133 (28.3%)	22 (26.5%)		81 (25.4%)	43 (25.9%)		32 (24.4%)	24 (17.6%)	
	HFpEF	101 (21.4%)	9 (10.8%)		72 (22.6%)	15 (9.0%)		8 (6.1%)	10 (7.4%)	
Medications	ACEI	26 (5.5%)	2 (2.4%)	0.310 ^b^	22 (6.9%)	3 (1.8%)	0.081 ^b^	9 (6.9%)	3 (2.2%)	0.083 ^b^
	ARB	194 (41.3%)	33 (39.8%)	0.855	136 (42.6%)	64 (38.6%)	0.525	59 (45.0%)	49 (36.0%)	0.139
	ARNI	237 (50.4%)	29 (34.9%)	0.034 *	162 (50.8%)	69 (41.65)	0.123	61 (46.6%)	65 (47.8%)	0.800
	Beta-blocker	417 (88.7%)	73 (88.0%)	0.922	288 (90.3%)	144 (86.7%)	0.343	118 (90.1%)	118 (86.8%)	0.362
	SGLT2	104 (22.1%)	15 (18.1%)	0.517	78 (24.5%)	28 (16.9%)	0.140	36 9(27.5%)	21 (15.4%)	0.021 *
	MRAs	318 (67.7%)	54 (65.1%)	0.705	220 (69.0%)	106 (63.9%)	0.372	86 (65.6%)	92 (67.6%)	0.728
	Diuretics	394 (83.8%)	62 (74.7%)	0.044 *	283 (88.7%)	127 (76.5%)	0.004 *	119 (90.8%)	105 (77.2%)	0.002 *
	Statins	223 (47.4%)	44 (53.0%)	0.417	142 (44.5%)	91 (54.8%)	0.087	57 (43.5%)	72 (52.9%)	0.138
	DM drugs ^c^	89 (18.9%)	14 (16.9%)	0.675	67 (21.0%)	24 (14.5%)	0.181	25 (19.1%)	24 (17.6%)	0.828
	Aspirin	149 (31.7%)	32 (38.6%)	0.340	104 (32.6%)	56 (33.7%)	0.833	39 (29.8%)	48 (35.3%)	0.335
	Clopidogrel	76 (16.2%)	15 (18.1%)	0.684	55 (17.2%)	26 (15.7%)	0.714	22 (16.8%)	22 (16.2%)	0.956
	Warfarin	31 (6.6%)	4 (4.8)	0.772 ^b^	18 (5.6%)	12 (7.2%)	0.553	7 (5.3%)	10 (7.4%)	0.472
	P2Y12-ADP	9 (1.9%)	3 (3.6%)	0.621 ^b^	8 (2.5%)	3 (1.8%)	1.000 ^b^	2 (1.5%)	4 (2.9%)	0.684
Survive	Death	22 (4.7%)	10 (12.1%)	0.009 *	11 (3.4%)	21 (12.7%)	<0.001 *	6 (4.6%)	20 (14.7%)	0.005 *

Data are the mean ± standard deviation or No. (percentage); ACEI, angiotensin-converting enzyme inhibitor; ARB, angiotensin receptor blocker; ARNI, angiotensin receptor–neprilysin inhibitor; BMI, body mass index; COPD, chronic obstructive pulmonary disease; Cr, creatinine; HbA1C, glycated hemoglobin; HFrEF, HF with reduced ejection fraction; HFmrEF, HF with mid-range ejection fraction; HFpEF, HF with preserved ejection fraction; LDL, low-density lipoprotein cholesterol; LVEF, left ventricular ejection fraction; MRAs, mineralocorticoid receptor antagonist; NYHA, New York Heart Association classification for heart failure; P2Y12-ADP, P2Y12-ADP receptor antagonists; SGLT2, sodium–glucose co-transporter 2 inhibitor; TC, total cholesterol; * *p* < 0.05, ^a^ All the comparisons between two groups were carried out by independent *t*-test, except for comparisons of categorical variables between the two groups were conducted by Chi-squared test or ^b^ Fisher’s Exact test. ^c^ Except for SGLT2.

**Table 2:** Variables of cardiopulmonary exercise testing according to outcome group.

Variables	Within 3 Months			Within 1 Year			Within 5 Years		
	Adverse Event-Free Group (*n* = 470)	Adverse Event Group (*n* = 83)	*p* Value	Adverse Event-Free Group (*n* = 319)	Adverse Event Group (*n* = 166)	*p* Value	Adverse Event-Free Group (*n* = 131)	Adverse Event Group (*n* = 136)	*p* Value
Resting heart rate (bpm)	76.2 ± 13.8	74.7 ± 12.8	0.345	76.1 ± 14.1	74.6 ± 12.8	0.241	77.0 ± 15.2	74.8 ± 11.9	0.182
Resting systolic BP (mmHg)	118.6 ± 21.1	114.2 ± 22.3	0.086	119.2 ± 20.2	111.6 ± 20.9	<0.001 *	118.1 ± 19.2	112.0 ± 20.9	0.015 *
Resting diastolic BP (mmHg)	69.7 ± 11.7	68.8 ± 11.9	0.490	70.4 ± 11.2	67.5 ± 12.5	0.008 *	70.7 ± 11.6	68.6 ± 12.8	0.159
Peak systolic BP (mmHg)	146.6 ± 30.2	143.0 ± 30.3	0.323	148.3 ± 28.7	139.3 ± 31.0	0.002 *	148.3 ± 27.6	138.0 ± 28.5	0.003 *
Peak diastolic BP (mmHg)	75.0 ± 13.8	73.5 ± 13.9	0.363	75.5 ± 13.3	72.6 ± 13.8	0.024 *	75.6 ± 13.7	72.4 ± 13.5	0.052
Chronotropic index (%)	41.1 ± 25.4	40.7 ± 23.6	0.878	41.0 ± 25.6	37.9 ± 22.2	0.177	36.8 ± 24.4	34.3 ± 22.6	0.407
Peak respiratory exchange ratio	1.1 ± 0.1	1.1 ± 0.1	0.819	1.1 ± 0.1	1.1 ± 0.1	0.579	1.1 ± 0.1	1.1 ± 0.1	0.194
Peak MET	3.6 ± 1.1	3.2 ± 1.0	0.010 *	3.6 ± 1.2	3.2 ± 1.0	<0.001 *	3.3 ± 1.0	2.9 ± 0.9	<0.001 *
Maximal Watt	55.1 ± 28.7	50.5 ± 26.5	0.174	55.1 ± 28.2	50.0 ± 27.2	0.057	49.0 ± 23.3	42.2 ± 22.1	0.015 *
Predicted peak VO2%	51.9 ± 19.2	50.5 ± 26.5	0.038 *	53.1 ± 20.3	46.0 ± 15.1	<0.001 *	47.7 ± 18.2	42.6 ± 14.7	0.013 *
Minute ventilation (L/min)	35.4 ± 12.3	35.7 ± 14.7	0.812	35.5 ± 12.2	35.0 ± 13.2	0.667	33.8 ± 10.9	32.9 ± 12.6	0.506
VE/VCO2 slope	36.1 ± 14.0	39.8 ± 12.5	0.026 *	36.1 ± 13.9	39.2 ± 14.8	0.028 *	37.2 ± 12.0	42.2 ± 15.6	0.004 *
Peak rate pressure product	16,427 ± 5345	15,753 ± 5465	0.294	16,563 ± 5144	15,079 ± 5262	0.003 *	16,179 ± 4850	14,528 ± 5123	0.008 *
OUES 100	1.2 ± 0.6	1.1 ± 0.6	0.033 *	1.2 ± 0.5	1.1 ± 0.6	0.080	1.1 ± 0.5	1.0 ± 0.5	0.021 *
OUES 100/BSA	0.7 ± 0.3	0.6 ± 0.3	0.048 *	0.7 ± 0.3	0.6 ± 0.3	0.011 *	0.6 ± 0.2	0.6 ± 0.2	0.004 *
6MWT (m)	328.9 ± 122.2	286.0 ± 125.1	0.004 *	332.2 ± 123.2	286.2 ± 119.6	<0.001 *	297.9 ± 119.3	257.7 ± 118.8	0.008 *
HR after 6MWT (bpm)	91.4 ± 14.7	91.3 ± 15.4	0.934	90.8 ± 14.7	90.2 ± 14.6	0.693	90.8 ± 15.0	87.7 ± 14.8	0.097
Borg scale after 6MWT	3.5 ± 1.7	3.9 ± 1.6	0.057	3.5 ± 1.7	3.8 ± 1.6	0.073	3.6 ± 1.6	4.0 ± 1.7	0.075

6MWT, 6-min walking test; AT, anaerobic threshold; BP, blood pressure; Chronotropic index ((peak heart rate-resting heart rate)/((220 − age) − resting heart rate)) × 100%; MET, metabolic equivalent; Predicted peak VO2%, percentage of measured peak oxygen consumption to estimated peak oxygen consumption; VE/VCO2 slope, minute ventilation to carbon dioxide production slope; OUES 100, oxygen uptake efficiency slope calculated from data of the whole exercise duration; BSA, body surface area. All the comparisons between two groups were conducted by independent *t*-test. * *p* < 0.05.

**Table 3:** Optimal cut-off points and related diagnostic value by receiver operating characteristic curve analysis.

	Cut-Off Point	Sensitivity	Specificity	AUC	*p* Value
Within 3 months					
Peak MET	1.85	0.974	0.032	0.428	0.043 *
Predicted peak VO2%	35.38	0.821	0.202	0.424	0.033 *
VE/VCO2 slope	38.95	0.538	0.698	0.607	0.003 *
OUES 100	3.35	0.013	0.998	0.419	0.022 *
OUES 100/BSA	1.5094	0.013	0.998	0.429	0.047 *
6MWT (m)	49.65	0.987	0.018	0.408	0.010 *
Within 1 year					
Resting systolic BP (mmHg)	157.50	0.058	0.970	0.385	<0.001 *
Resting diastolic BP (mmHg)	88.50	0.077	0.943	0.431	0.015 *
Peak systolic BP (mmHg)	190.50	0.096	0.939	0.399	<0.001 *
Peak diastolic BP (mmHg)	103.50	0.045	0.970	0.439	0.033 *
Peak MET	1.00	1.00	0.003	0.399	<0.001 *
Predicted peak VO2%	11.52	0.583	0.698	0.399	<0.001 *
VE/VCO2 slope	38.95	0.455	0.703	0.583	0.004 *
Peak rate pressure product	27,527	0.045	0.970	0.409	0.001 *
OUES 100/BSA	1.4951	0.013	1.000	0.416	0.003 *
6MWT (m)	28.00	0.994	0.010	0.408	0.001 *
Within 5 years					
Resting systolic BP (mmHg)	151.5	0.065	0.975	0.389	0.007 *
Peak systolic BP (mmHg)	199.50	0.049	0.975	0.388	0.003 *
Peak MET	1.00	1.000	0.008	0.391	0.003 *
Maximal Watt	7.00	0.992	0.017	0.416	0.024 *
Predicted peak VO2%	30.55	0.846	0.167	0.424	0.039 *
VE/VCO2 slope	37.35	0.577	0.633	0.621	0.001 *
Peak rate pressure product	23,550	0.073	0.958	0.399	0.006 *
OUES 100	1.90	0.073	0.958	0.410	0.016 *
OUES 100/BSA	0.12	0.992	0.008	0.410	0.016 *
6MWT (m)	44.15	0.976	0.033	0.414	0.021 *

6MMWT, 6-min walking test; AT, anaerobic threshold; BP, blood pressure; MET, metabolic equivalent; Predicted peak VO2%, percentage of measured peak oxygen consumption to estimated peak oxygen consumption; VE/VCO2 slope, minute ventilation to carbon dioxide production slope; OUES 100, oxygen uptake efficiency slope calculated from data of the whole exercise duration; BSA, body surface area. * *p* < 0.05.

**Table 4:** Predictive measures for three-month, one-year, and five-year major adverse cardiovascular events in patients who survived acute decompensated heart failure.

Variable	*p* Value-Multivariate	Hazard Ratio-Multivariate ^a^	95% CI
Within 3 months			
VE/VCO2 slope ≤ 38.95	<0.001 *	1.000	
VE/VCO2 slope > 38.95		2.488	1.607–3.852
Within 1 year			
VE/VCO2 slope ≤ 38.95	<0.001 *	1.000	
VE/VCO2 slope > 38.95		1.807	1.325–2.464
Within 5 years			
VE/VCO2 slope ≤ 37.35	0.002 *	1.000	
VE/VCO2 slope > 37.35		1.749	1.233–2.483

VE/VCO2 slope, minute ventilation to carbon dioxide production slope; ^a^ adjusted for age and gender; * *p* < 0.05.

## Data Availability

Individual participant data that underlie the results reported in this article, after deidentification, might be shared. Proposals should be directed to gabbrile@vghks.gov.tw for application.
